# *Trypanosoma cruzi* Coaxes Cardiac Fibroblasts into Preventing Cardiomyocyte Death by Activating Nerve Growth Factor Receptor TrkA

**DOI:** 10.1371/journal.pone.0057450

**Published:** 2013-02-21

**Authors:** Daniel Aridgides, Ryan Salvador, Mercio PereiraPerrin

**Affiliations:** 1 Graduate Program in Immunology, Sackler School of Graduate Biomedical Sciences, Tufts University, Boston, Massachusetts, United States of America,; 2 Department of Pathology, Tufts University School of Medicine, Boston, Massachusetts, United States of America; National Institutes of Health, United States of America

## Abstract

**Rationale:**

Cardiomyocytes express neurotrophin receptor TrkA that promotes survival following nerve growth factor (NGF) ligation. Whether TrkA also resides in cardiac fibroblasts (CFs) and underlies cardioprotection is unknown.

**Objective:**

To test whether CFs express TrkA that conveys paracrine signals to neighbor cardiomyocytes using, as probe, the Chagas disease parasite *Trypanosoma cruzi*, which expresses a TrkA-binding neurotrophin mimetic, named PDNF. *T cruzi* targets the heart, causing chronic debilitating cardiomyopathy in ∼30% patients.

**Methods and Results:**

Basal levels of TrkA and TrkC in primary CFs are comparable to those in cardiomyocytes. However, in the myocardium, TrkA expression is significantly lower in fibroblasts than myocytes, and vice versa for TrkC. Yet *T cruzi* recognition of TrkA on fibroblasts, preferentially over cardiomyocytes, triggers a sharp and sustained increase in NGF, including in the heart of infected mice or of mice administered PDNF intravenously, as early as 3-h post-administration. Further, NGF-containing *T cruzi-* or PDNF-induced fibroblast-conditioned medium averts cardiomyocyte damage by H_2_O_2_, in agreement with the previously recognized cardioprotective role of NGF.

**Conclusions:**

TrkA residing in CFs induces an exuberant NGF production in response to *T cruzi* infection, enabling, in a paracrine fashion, myocytes to resist oxidative stress, a leading Chagas cardiomyopathy trigger. Thus, PDNF-TrkA interaction on CFs may be a mechanism orchestrated by *T cruzi* to protect its heart habitat, in concert with the long-term (decades) asymptomatic heart parasitism that characterizes Chagas disease. Moreover, as a potent booster of cardioprotective NGF *in vivo*, PDNF may offer a novel therapeutic opportunity against cardiomyopathies.

## Introduction

Activation of receptor tyrosine kinases TrkA, TrkB and TrkC by the neurotrophins (NTs) nerve growth factor (NGF), brain-derived neurotrophic factor (BDNF) and NT-3, respectively, is important for proper central nervous system (CNS) development and repair [1], [2]. Conversely, malfunction of Trk receptor signaling in the brain exacerbates Huntington, Alzheimer and other neurodegenerative diseases, a process that can be reversed by NT administration [3], [4].

Trk signaling is also critical for cardiovascular development, as exemplified by mice bearing targeted deletion of NT-3 or TrkC, which exhibit early postnatal mortality and defects in cardiac septation, valvulogenesis, and truncal formation [5], [6]. In adulthood, Trk signaling, particularly the NGF/TrkA axis is critical for maintenance of cardiac nerves [7], [8], [9], and survival of cardiac myocytes [10] and endothelial cells [11]. NGF gene therapy accelerates cardiac repair following myocardial infarction [12] or type 1 diabetes [13], further establishing TrkA as cardioprotective. The known source of NGF that triggers cardiomyocyte survival is the cardiomyocyte itself – hence the dogma that NGF acts on cardiomyocytes through an autocrine input [10], [12]. Whether cardiac fibroblasts (CFs) communicate with cardiomyocytes via NTs to regulate cardiac function and response to stressors remains a mystery, however.

CFs outnumber cardiomyocytes in the adult heart and form a dense network of cells surrounding clusters of myocytes. Thus, fibroblasts are strategically located to influence cardiomyocyte function and response to injury through direct cell-cell interactions and paracrine communication. Although CFs have long been recognized as a major source of cardiac nonbasement membrane extracellular matrix [14], [15], recent evidence indicates that fibroblasts regulate cardiomyocyte phenotype and vice versa, under physiological conditions and in response to injury, through gap junction-based direct cell-cell interaction, and by releasing growth factors and cytokines such as transforming growth factor-β (TGF-β), fibroblast growth factor-2 (FGF-2), interleukin-6 (IL-6) [16], [17], [18]. CFs express receptors for those growth factors and cytokines, several in common with cardiomyocytes and some more abundant in the fibroblasts, like angiotensin receptors, which link the renin-angiotensin-aldosterone system to myocardial and extracellular matrix remodeling and which convey paracrine signals to cardiomyocytes via TGF-β secretion [19]. CFs are the subject of current great excitement as they can be reprogrammed into functional cardiomyocytes using specific transcription factors [20], [21], [22] or micro-RNAs [23], and therefore could be critical for efficient restoration of necrotic cardiomyocytes in cardiomyopathies.

*Trypanosoma cruzi* causes incurable Chagas disease that afflicts millions of people worldwide, mostly in Latin America. Chagasic patients develop cardiomyopathy, which manifests clinically as arrhythmias, right bundle blocks, apical aneurysms, heart failure and sudden death. Patients may also display gut disturbances that lead to megacolon/megaesophagus. However, detrimental heart and/or gut parasitism occurs in a minority (∼30 to 40%) of patients, and even so, only after years or decades of asymptomatic and pathology-free infection [24], [25], raising the possibility that *T cruzi* orchestrates mutually beneficial protective mechanisms in infected tissues. This hypothesis is reinforced by the fact that, if only 40 to 30% patients develop pathology, then most chagasic patients (60–70%) remain asymptomatic and without detectable cardiac and gut pathology (indeterminate phase) for life.

One mechanism that might explain *T cruzi* manipulation of tissue survival events results from the trophic actions of parasite-derived neurotrophic factor (PDNF) {Chuenkova, 2011 #2427}. This *T cruzi* outer membrane protein mimics NTs by interacting with TrkA and TrkC, driving entry into cells of the nervous system while concomitantly activating anti-apoptotic signaling [26], [27], [28], akin to pro-survival actions of endogenous NGF and NT-3 [1], [2]. PDNF, which we originally identified in *T cruzi* by its neuraminidase activity [29], also transfers sialic acid to β-Gal acceptors (*trans*-sialidase activity) [30]. However, the Trk-binding activity of PDNF is independent of sialic acid recognition because point mutations in the protein reduce neuraminidase and trans-sialidase activities with no impairment in neurotrophic properties [31] and because the properties of a PDNF-modeled synthetic peptide (21-mer), which reproduces biological activities and signaling of parental PDNF [32]. Moreover, PDNF, after phosphorylation by prosurvival Akt kinase, promotes cell survival in the cell cytosol where sialyl-glycoconjugates are absent [33].

Here we report that CFs express NTs and Trk receptors, and that one of the NT/Trk pairs, NGF/TrkA, plays role in fibroblast-cardiomyocyte communication in response to *T cruzi* and its PDNF.

## Methods

### Ethics statement

All mouse work was approved by the Institutional Animal Care and Use Committee at Tufts University School of Medicine (Protocol B2010-32) and by the Department of Laboratory Animal Medicine of Tufts University and Tufts Medical Center.

### Immunofluorescence – frozen sections

Adult mouse hearts were fixed in 4% paraformaldehyde overnight, dehydrated in 15% and 30% sucrose solutions for 24 hours each, then mounted in OCT solution and sectioned. Sections were permeabilized in 0.1% Triton X-100 for 5 minutes, blocked with PBST/10% FCS overnight at 4°C, then incubated with primary antibody in PBST/5% FCS overnight at 4°C. Secondary antibody was incubated at room temperature for 2 hours. Sections were washed three times with PBST between each step. Images were acquired with Nikon Brightroom Elements software and analyzed by a custom script using NIH Image J software to bin data based on Trk fluorescence levels (Red) and computing mean myosin heavy chain (MHC) and vimentin intensities. Antibodies with concentrations and sources are listed in Table S1.

### Immunofluorescence – primary cultures

As above except primary cultures were washed with PBS, fixed in 4% paraformaldehyde for 20 minutes, permeabilized, blocked, and stained. Some cells were stained with 4',6-diamidino-2-phenylindole (DAPI) to visualize nuclei. Images were acquired with SPOT imaging system and prepared in Adobe Photoshop.

### Primary cardiomyocyte and cardiac fibroblast isolation

We used a slightly modified version of the procedure described by Sreejit et al [34]. Neonatal C57BL/6 mice (1–3 days old) were sacrificed by decapitation, hearts were dissected and washed twice in PBS, and kept in 20 mM HEPES, 130 mM NaCl, 1 mM NaH_2_PO_4_, 4 mM glucose, 3 mM KCl, pH 7.6 for 10 minutes on ice. Hearts were then minced and digested in 0.25% Trypsin-EDTA (Gibco) 3–4 times at 37°C with periodic mixing. Digests were pooled, stopped with DMEM/10% FCS, passed through a 100 µm cell strainer, centrifuged, then plated for 3 hours on 1% gelatin-coated plates in DMEM (Gibco)/F12 Ham's (Sigma) 50∶50, 20% FCS (PAA), 5% horse serum (PAA), 2 mM L-glutamine (Gibco), 0.1 mM nonessential amino acids (Gibco), 3 mM sodium pyruvate (Gibco), and 1 µg/ml bovine insulin (Sigma) with 1x penicillin-streptomycin (Gibco). After three hours, non-adherent cells (cardiomyocytes) were removed and plated onto gelatin-coated plates while adherent cells (cardiac fibroblasts) were grown in DMEM/10% FCS until needed. Purity of preparations was monitored visually and/or by immunofluorescence and was routinely >90%.

### *In vitro* Infection with *T cruzi*

*T cruzi* (Tulahuén strain) were harvested from supernatants of infected monolayers of Vero cells where they were maintained. Supernatants were centrifuged at 960 rpm in a tabletop centrifuge to remove any detached cells then parasites were pelleted at 2400 rpm and resuspended in DMEM/0.1% FCS. Parasites were counted in a hemacytometer and added to primary cardiomyocytes or cardiac fibroblasts as well as the H9c2 cardiomyocyte cell line (ATCC). Supernatants of infected cells were cleared by centrifugation, filtered, and frozen or analyzed immediately by ELISA. Monolayers of cells were lysed in Trizol reagent (Invitrogen) for RNA isolation.

### Elisa

Wells of a 96-well plate were coated overnight with NGF capture antibody (Chemicon AB1528, 1∶1000) in coating buffer (50 mM NaHCO_3_/Na_2_CO_3_, pH 9.6, 0.02% NaN_3_). Wells were blocked in PBST/5% BSA, incubated with supernatants followed by a second NGF antibody (Chemicon AB1526, 1∶1000) then alkaline-phosphatase conjugated detection antibody (Sigma A3687, 1∶1000). All incubations were 90 min at room temperature and wells were washed three times with PBST in between each step. Wells were then incubated with colorigenic AP substrate (Sigma N9389, 1 mg/mL in 100 mM Glycine, 1 mM MgCl_2_, 1 mM ZnCl_2_ pH 10.4). NGF concentrations were calculated relative to a standard curve (NGF, Sigma N0513).

### Quantitative Reverse-Transcriptase Polymerase Chain Reaction

RNA was isolated from Trizol lysates of cell monolayers according to manufacturer instructions. cDNA was synthesized using Quantitect reverse transcription kit (Qiagen) according to manufacturer instructions. Trk and neurotrophin transcripts were amplified using specific primers and quantified relative to HPRT using SYBR Green (Qiagen). Primer sequences are listed in Table S2.

### Neurite extension assay

PC12 rat pheochromocytoma cells (a gift from Dr. Lloyd Greene, Columbia University [35]) were plated on 96-well plates, serum-starved overnight in 0.1% FCS, then incubated with media conditioned by cardiac fibroblasts infected with *T cruzi*. After 24 hours, cells were fixed in methanol, stained with Diff Quik stain and counted. In parallel, conditioned media from infected CF was pre-incubated with a neutralizing NGF antiserum (Chemicon AB1528, 1∶200) for 30 minutes at room temperature or with normal sheep serum (Chemicon NS114-nc). Neurite bearing (possessing at least one neurite of one cell-body length or greater) cells were quantified by optical microscopy (>200 cells/well).

### Lentiviral shRNA knockdown

Lentiviral particles encoding shRNA constructs against TrkA and TrkC were generated in HEK 293 cells and frozen at −80°C until needed in accordance to the instructions of the manufacturer (Open Biosystems). Knockdown conditions were optimized using immunofluorescence to measure Trk expression on the primary cultures of cardiac cells. 40 µL lentiviral particles were added per well of a 24-well plate and cells were cultured for 6 days to allow for expression of constructs.

### sPDNF purification

sPDNF was originally cloned from the Silvio X-10/4 strain of *T cruzi* (GenBank accession number AJ002174), expressed in BL21(DE3) bacteria, and purified by Ni-affinity chromatography, as described previously [36]. sPDNF migrates on SDS-PAGE as a single or double band of MW 68 kDa. If isolated sPDNF contained extra bands, it was re-purified on the same Ni-affinity gels to give rise to homogeneously pure recombinant PDNF. Protein was quantified by scanning densitometry of Coomassie-stained SDS-PAGE gels. sPDNF leter-sterilized (0.22 µ) and kept at 4°C at ∼ g/ml PBS (0.01 M phosphate buffered saline, pH 7.2); under these conditions, sPDNF keeps Trk-binding activity for many months.

### Mouse experiments

#### T cruzi infection

*T cruzi* from supernatants of infected Vero cell monolayers were isolated as described above, washed twice by resuspension in PBS and centrifugation, then counted and diluted in PBS to 1.67×10^5^/mL and kept on ice. 6–8 week old female C57BL/6 mice (Jackson Laboratories) were anesthetized by isoflurane inhalation, then injected with 30 µL *T cruzi* (5000/mouse) into the left hind footpad. At the indicated timepoints, mice were sacrificed by CO_2_ asphyxiation and cervical dislocation, a hole was cut in the right atrium, 5 mL PBS was perfused via the left ventricle, and hearts were flash frozen in liquid nitrogen and stored at −80°C or fixed in 4% paraformaldehyde for frozen sections. RNA and DNA were isolated from frozen hearts via Trizol (Invitrogen) extraction according to manufacturer instructions. Parasitemia was evaluated by optical microscopy of blood smears and heart parasite burden was measured by qPCR of a conserved microsatellite DNA sequence in *T cruzi* according to the procedure of Cummings and Tarleton [37], as previously described [27], [38].

NGF transcript levels were assessed by qPCR of heart cDNA and displayed by the 2^−ddCt^ method using HPRT as an internal control and time-0 as the experimental control. NGF protein levels at day 20 post-infection were assessed by staining frozen sections with NGF antiserum and quantifying signal with ImageJ.

#### sPDNF in vivo administration

sPDNF was diluted in PBS and injected (200 µl) via tail vein into 6–8 week old female C57BL/6 mice. 25 µg sPDNF was administered at 0, 3, and 24 hrs (2 day timepoint) or 0, 3, 24, 48, 72, and 96 hrs (6 day timepoint). Injection of PBS alone served as a negative control. Mice were perfused with PBS as above and hearts were separated into atria and ventricles and either flash frozen in liquid nitrogen and stored at −80°C or fixed in 4% paraformaldehyde for frozen sections.

### *Trans*-sialidase activity assay

*Trans-*sialidase activity in tissue lysates was measured by homogenizing tissue at 100 mg/mL in lysis buffer (50 mmol/L Tris-HCl pH 7.2, 0.5 mol/L NaCl, 1% Triton X-100, 1 mmol/L PMSF, 1x protease inhibitor cocktail (Roche)). Enzymatic activity was measured by catalysis of sialic acid from FCS to the ^14^C-labelled acceptor, N-acetyllactosamine (Sigma) as previously described [36]. Tissue concentrations of sPDNF were calculated from a standard curve obtained by spiking control tissues (where *trans-*sialidase activity is absent) with known concentrations of sPDNF. Data are composite from two independent experiments.

### Cardiomyocyte survival assay

Primary cardiomyocytes were pre-treated with unconditioned (from control cardiac fibroblasts) or conditioned (from *T cruzi-*infected or sPDNF-administered cardiac fibroblast) media. CoM was centrifuged (2, 000×g, 10 min) and filter-sterilized (0.22 µ) prior to addition to the myocyte cultures. Cardiomyocytes were separately treated with DMEM/0.1% FCS unexposed to cells, with or without 50 ng/mL NGF. In parallel, conditioned media was preincubated with α-NGF serum or control normal serum. Cardiomyocytes were then subjected to 150 µM hydrogen peroxide treatment for 4 hours and stained with Hoechst 33342 and propidium iodide to visualize live/dead cells. Dead cardiomyocytes are seen a pink dots (the result of the merge of dark-red PI and blue Hoechst staining), while viable cardiomyocytes are stained blue by the vital dye Hoechst 33342 and no PI staining. At least 200 cells/well were counted per point.

### Statistics

Statistical tests were performed with GraphPad Prism 5 software using Student's t-test for comparing two values or ANOVA with Tukey or Dunnett's post-test for comparing three or more values. P values are as indicated in figures with p<0.05 considered statistically significant.

## Results

### In the Myocardium, TrkA is Expressed Preferentially in Cardiomyocytes and TrkC in Cardiac Fibroblasts

**Figure 1:**
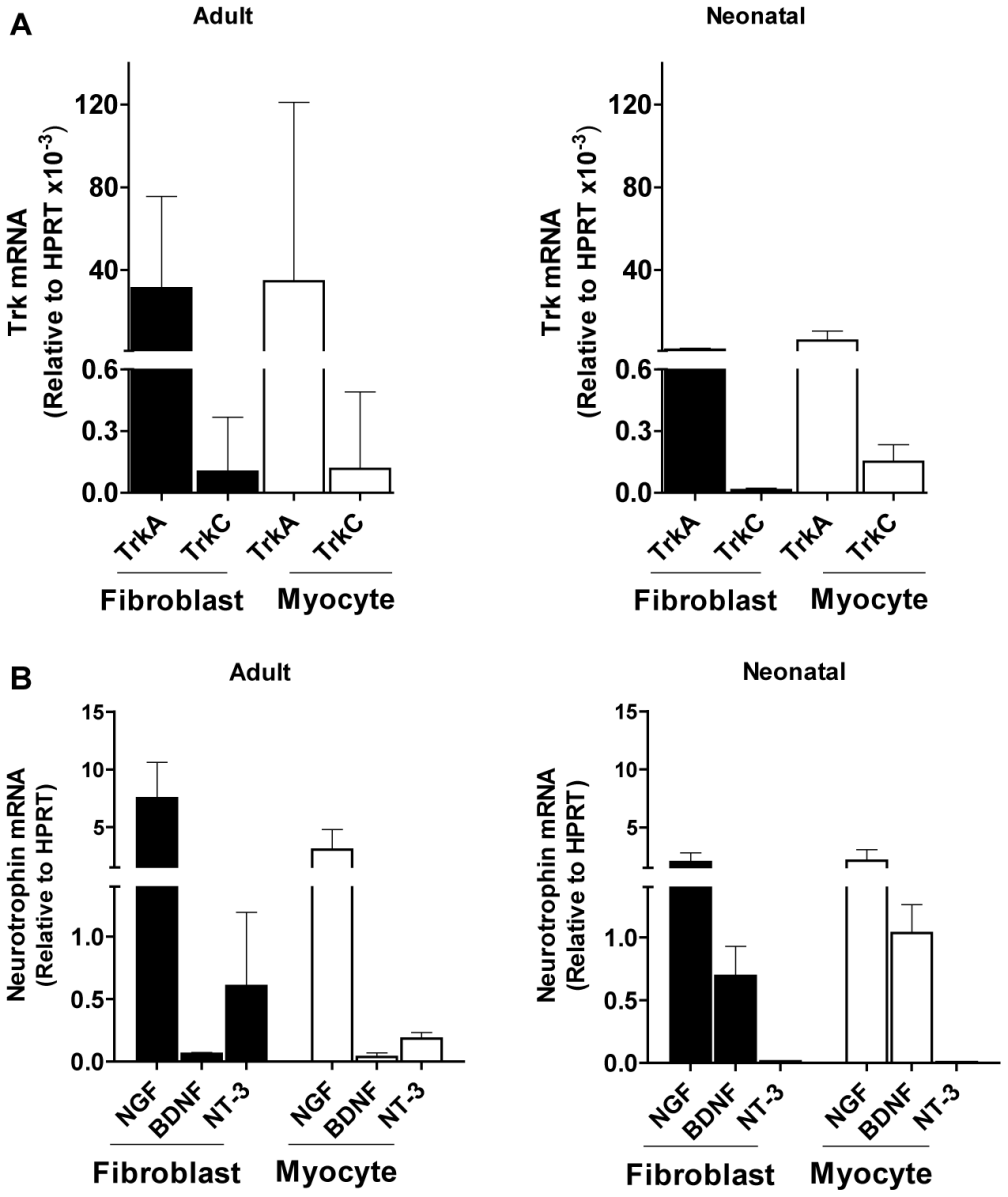
Expression of neurotrophin receptor and ligands in primary cultures of cardiac fibroblasts and myocytes. **A)** Primary cultures of cardiac fibroblasts express TrkA mRNA at levels higher than TrkC mRNA, comparable to the expression in cardiomyocytes. Cardiac fibroblasts and cardiomyocytes were isolated from adult female (**left**) or neonatal (**right**) C57BL/6 mice and their Trk transcripts quantified by qPCR. Data are combined from at least three separate experiments, each point in triplicate. **B)** Variable expression of neurotrophin mRNAs in primary cultures of adult or neonatal cardiac fibroblasts and myocytes. NGF, BDNF and NT-3 mRNAs were assessed by qPCR in primary cultures of adult female (**left**) or neonatal (**right**) cardiac fibroblasts (CF) and myocytes. Two technical replicates each on triplicate samples were normalized to HPRT and graphed as mean + s.d.

Early work by others demonstrated Trk receptors in cardiomyocytes [10], [39], [40]. We used real-time PCR to determine basal levels of Trk receptors in primary cultures of adult and neonatal mouse CFs. We find that fibroblasts express TrkA and TrkC transcripts, TrkA predominating over TrkC, at levels comparable to those in cardiomyocytes (Fig. 1A). Adult and neonatal cardiac cells exhibited similar patterns of Trk expression. Visualization of TrkA and TrkC by immunofluorescence in cardiac fibroblasts is consistent with the mRNA findings (data not shown).

In light of this Trk expression we next determined transcript levels of the Trk ligands NGF, BDNF, and NT-3. NGF was most highly expressed in both adult and neonatal cardiac fibroblasts and myocytes, however BDNF transcripts were more prevalent in neonatal cells while NT-3 transcripts increased in adult mice (Fig. 1B). Dissociation curves and agarose gel analysis of qPCR products demonstrated the homogeneity of amplicons (Fig. S1).

**Figure 2:**
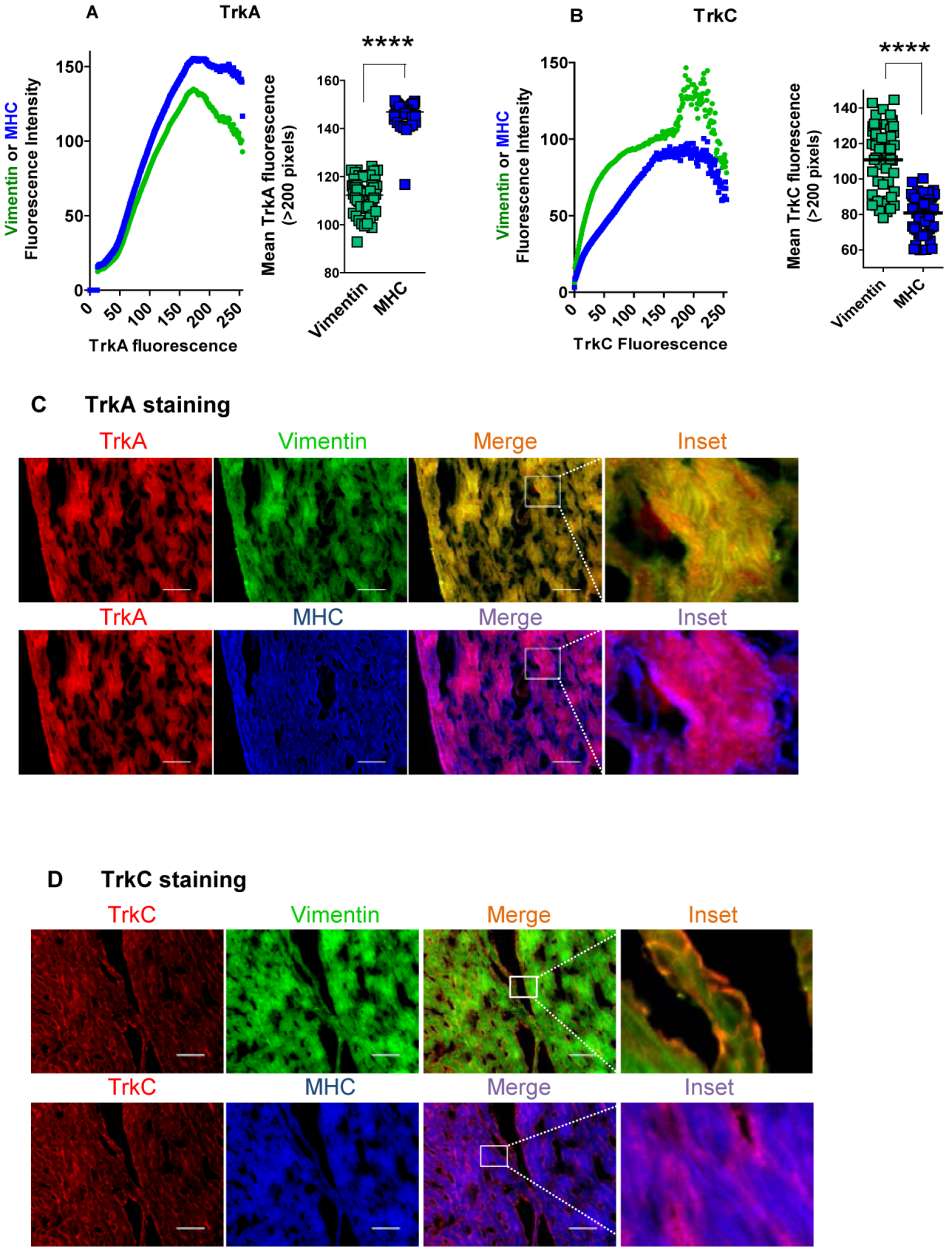
Cardiomyocytes preferentially expresses TrkA and cardiac fibroblasts TrkC. **A**) TrkA levels are significantly higher in cardiomyocytes than in cardiac fibroblasts in tissue sections of the heart. **Left panel** shows the quantification of TrkA, vimentin (cardiac fibroblast marker), and myosin heavy chain (MHC, cardiomyocyte marker) identified by immunofluorescence on sections of C57BL/6 mouse hearts. Vimentin and MHC staining is plotted as function of TrkA fluorescence showing that TrkA is expressed by both cardiac fibroblasts and myocytes and that expression is higher in the myocytes. **Right panel** is a plot of vimentin and MHC mean fluorescence on pixels where TrkA is >200 (n = 3); ****, p<0.0001. **B**) TrkC levels are significantly higher in cardiac fibroblasts than on cardiomyocytes in tissue sections of the heart. **Left panel** shows the quantification of TrkC, vimentin, and MHC identified by immunofluorescence on sections of C57BL/6 mouse hearts, showing a preferential association of TrkC with cardiac fibroblasts. **Right panel** is a plot of vimentin and MHC mean fluorescence on pixels where TrkC is >200 (n = 3);****, p<0.0001.**C**) Visualization of the preferential expression of TrkA on cardiomyocytes in the heart. Representative images from the results presented in (**A**) above. Note preferential merge of TrkA and cardiomyocyte staining, scale bar = 100 µm. **D**) Visualization of the preferential expression of TrkC in cardiac fibroblasts in the heart. Representative images from the results presented in (**B**) above. Of note, TrkC staining merges preferentially in vimentin-stained cardiac fibroblasts, typically surrounding cardiomyocyte bundles, scale bar = 100 µm.

To determine TrkA and TrkC protein levels in the myocardium, we reacted heart (ventricle) tissue sections with antibodies against receptors TrkA or TrkC and against markers of CF (vimentin) and cardiomyocytes (myosin heavy chain, MHC), followed by fluorescent-labeled secondary antibodies. Then, we quantified specific fluorescence pixels in the receptors and markers using NIH ImageJ software, revealing TrkA in both myocardial fibroblasts and myocytes but more prominently in the myocytes (Fig. 2A), and TrkC predominating in CFs (Fig. 2B). In validation of this expression pattern, visualization of stained cardiac tissue sections shows that, although TrkA merges with both MHC and vimentin, it prevails with the cardiomyocyte marker (Fig. 2C), quite the contrary of TrkC, which merges primarily with the fibroblast marker vimentin (green) (Fig. 2D).

### *T cruzi* Infection of Cardiac Fibroblasts, but not Cardiomyocytes, Triggers a Robust Expression of Bioactive NGF

Based on real-time PCR, basal level of NGF transcript in primary neonatal CFs is ∼3-fold higher than BDNF and ∼120-fold higher than NT-3 transcript (Fig. 1B), analogous to primary cardiomyocyte expression except for NT-3, which is not detectable in the myocytes (Fig. 1B). As *T cruzi* interacts with both TrkA [26] and TrkC [41], we tested whether NT expression in CFs and/or cardiomyocytes is altered in response to *T cruzi* infection.

**Figure 3:**
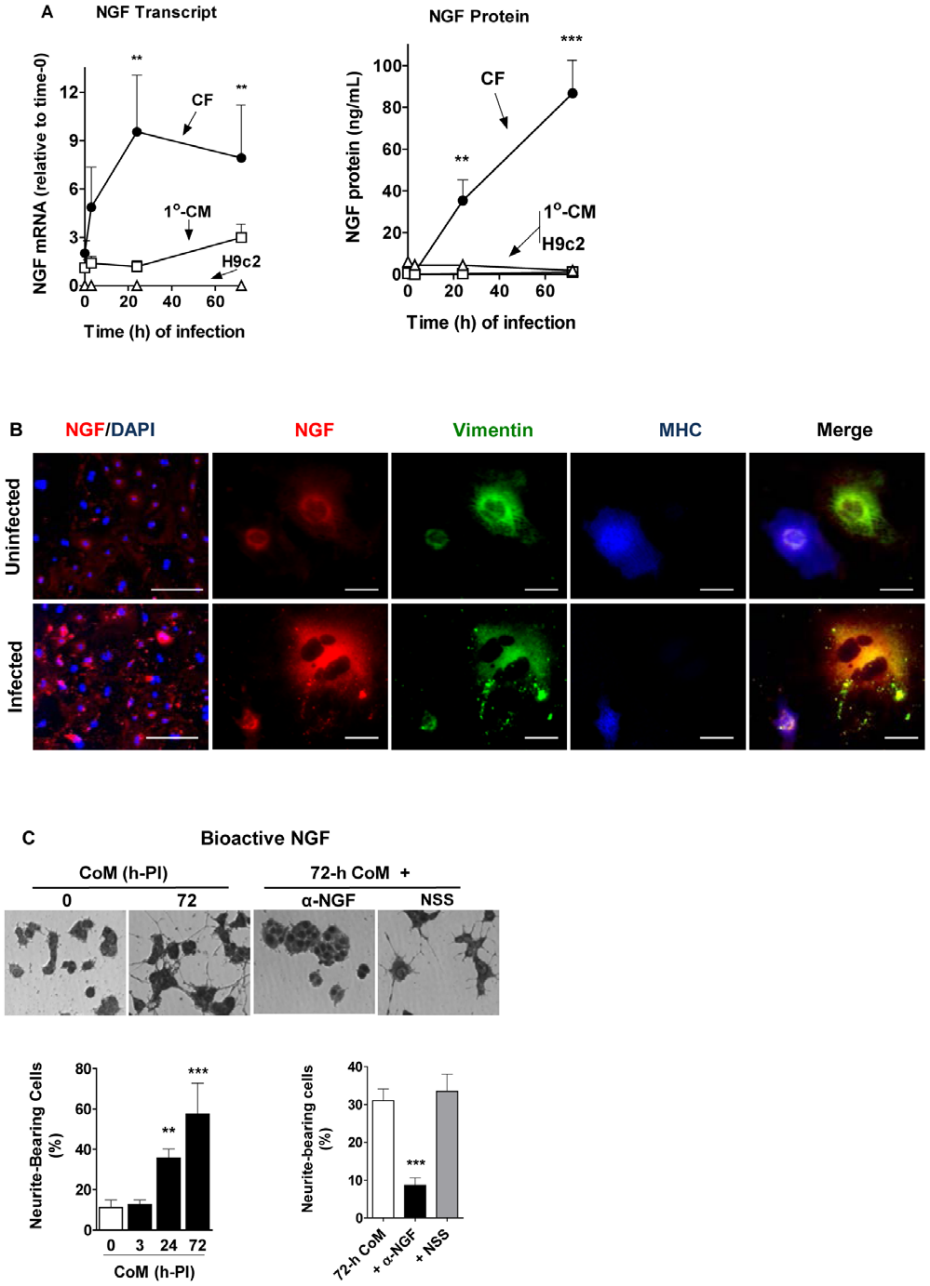
T cruzi infection upregulates NGF preferentially in primary cultures of cardiac fibroblasts compared to cardiomyocytes. A) T cruzi selectively upregulates NGF in cardiac fibroblasts. Primary cardiac fibroblasts (CFs) and cardiomyocytes (1°-CMs), and the cardiomyocyte cell line H9c2 were infected with T cruzi trypomastigotes (2×10^5^ per ml), and, at the indicated time points, their NGF mRNA was measured by qPCR (left panel). Similarly, culture supernatants were harvested and their NGF protein levels quantified by ELISA (right panel). Data are representative of three identical experiments; **, p<0.01, ***, p<0.001. B) Visualization of the preferential upregulation of NGF in cardiac fibroblast in T cruzi-infected co-culture of cardiac cells. Primary co-cultures (∼95% cardiac fibroblasts and 5% cardiomyocytes) were infected or not with 2×10^5^ T cruzi/mL for 24 h, then fixed. Leftmost panels: cells stained for NGF (red), and nuclei counterstained with DAPI (blue), scale bars = 100 µm. Right panels: high magnification of cells stained for NGF (red), vimentin (green), and MHC (blue), revealing upregulated NGF preferentially localized on cardiac fibroblasts in the T cruzi-infected co-cultures; scale bars = 10 µm.C) Cardiac fibroblasts secrete bioactive NGF in response to T cruzi infection. Primary cardiac fibroblasts were infected with T cruzi for 0 or 72 h and their conditioned media (CoM) were added to PC12 cell monolayers (left and center left panels). Note a robust neurite outgrowth produced by the 72-h CoM which was abolished by preincubating with a sheep antiserum against NGF (α-NGF) but not with normal sheep serum (NSS) (center right and right panels). Statistical significance is demonstrated in the bar-graphs, which represent the mean ± sd of >200 cells/well of triplicate wells, representative of three experiments; **, P<0.01; ***, P<0.001.

We find that *T cruzi-*infected CFs, but not cardiomyocytes, exhibit a dramatic increase in NGF expression. For example, NGF transcript in *T cruzi*-infected CFs increases by more than 9-fold at 24-h post-infection (PI) whereas NGF protein in the culture supernatants is boosted by more than 80-fold at 72-h PI (Fig. 3A). This response is in sharp distinction to the one produced by *T cruzi* infection of primary cardiomyocytes and of the cardiomyocyte cell line H9c2, which upregulate NGF only slightly and not at all, respectively (Fig, 3A). Early microarray studies showed that *T cruzi* infection of primary cardiomyocytes upregulate NGF mRNA [42]. Because BDNF and NT-3 transcripts, whether in CFs or myocytes, did not significantly change in response to *T cruzi* infection (data not shown), they are not studied further here.

Indirect immunofluorescence of cardiac fibroblasts/myocytes co-cultures validates the robust and cell-selective NGF upregulation (Fig. 3B, leftmost panels). Co-staining for NGF, vimentin and MHC confirms the preponderance of NGF in CFs as judged by the preferential merge of fluorescently labeled vimentin with NGF (Fig. 3B).

NGF secreted in response to *T cruzi* infection is bioactive because 1) conditioned medium (CoM) obtained from *T cruzi*-infected primary CFs at 72 h post-infection (PI), but not medium from uninfected cells (0-h CoM) strongly promotes neurite outgrowth in PC12 cells (Fig. 3C, left panels) in a time-of-infection dependent manner (Fig. 2C, left bar graph), and 2) an NGF anti-serum blocks the neurite-enhancement effect of active CoM nearly completely (Fig. 3C, right panels and bar graph).

### Targeting TrkA in Cardiac Fibroblasts for NGF Upregulation

**Figure 4:**
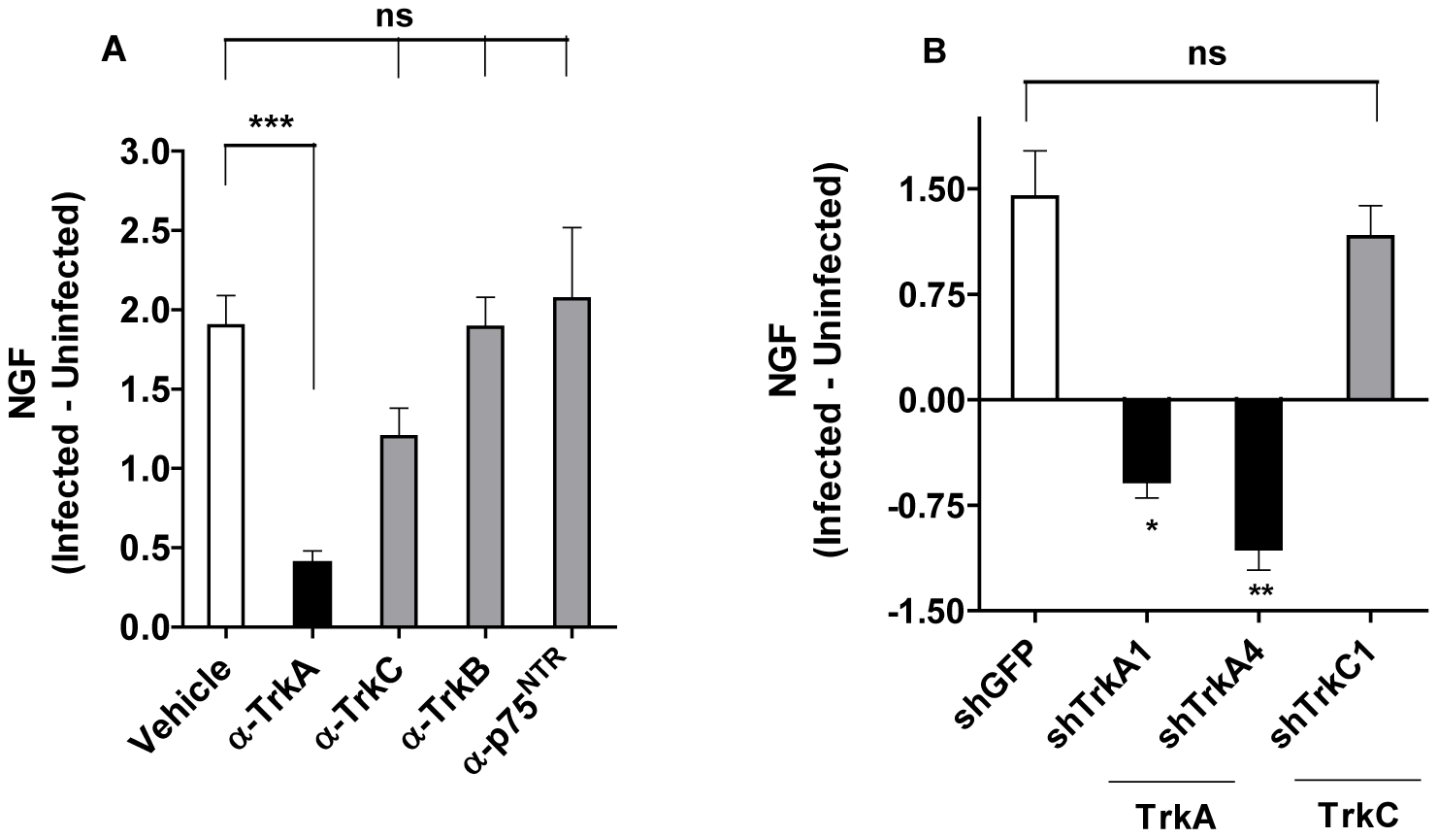
TrkA is targeted by T cruzi for upregulation of NGF in cardiac fibroblasts. **A**) Antibodies against TrkA, but not against TrkB, TrkC or pan-neurotrophin receptor p75 (p75^NTR^) significantly abrogate T cruzi-induced upregulation of NGF on cardiac fibroblasts. Primary cardiac fibroblasts were preincubated with the indicated antibodies (1 µg/ml), infected with T cruzi for 24 h, and the concentration of NGF in the culture overlays determined by ELISA. The results are the mean ± sd of triplicate points and represent the difference between NGF secreted by infected and uninfected cardiac fibroblasts; ***, P<0.001.**B**) shRNA against TrkA abrogates T cruzi-induced upregulation of NGF on cardiac fibroblasts. Cardiac fibroblasts were transfected with lentivirus encoding shRNA constructs against GFP, TrkA (two distinct vectors), or TrkC (one vector). Seven days later, fibroblasts were infected with T cruzi for 24 h, and NGF content in the culture overlays determined by ELISA. Results are the mean ± SEM of five separate experiments with similar results, and represent the difference between NGF secreted by infected and uninfected cardiac fibroblasts; *, P<0.05, **, P<0.005.

To determine whether *T cruzi* exploits fibroblast TrkA or TrkC, or both, to trigger NGF production, CFs were pre-incubated (0.5 h) with antibodies against the neurotrophin receptors prior to *T cruzi* infection (24 h). If *T cruzi* uses Trks to increase NGF expression, then neutralizing antibodies against relevant Trks should block the *T cruzi* action. We find that antibodies against TrkA (α-TrkA) significantly block secretion of NGF (∼80% inhibition, P<0.001) while antibodies against TrkC (α-TrkC) are less effective (∼36% inhibition, not statistically significant) (Fig. 4A). Further underlying the selectivity of TrkA blockage, antibodies against TrkB (α-TrkB) and against the pan-neurotrophin receptor p75^NTR^ (α-p75^NTR^), which do not interact with *T cruzi* [26], [28], exhibit no inhibitory activity. Therefore, these results suggest that *T cruzi* boosts NGF production in cardiac fibroblasts predominantly through TrkA recognition.

Experiments designed to reduce Trk expression with shRNA validate the conclusion from the antibody blocking experiments. We transfected CFs with lentivirus encoding shRNA constructs for control green fluorescence protein (GFP), TrkA (two distinct vectors), or TrkC (one vector) and infected the transfected cardiac fibroblasts with *T cruzi* for 24 h. Compared to GFP-transfected fibroblasts, the cell lines transfected with two shTrkA constructs, but not the cell line transfected with the TrkC mRNA silencer, selectively block NGF secretion in response to *T cruzi* infection (Fig. 4B).

### Selective Upregulation of NGF in Cardiac Fibroblasts in Chagasic Hearts

The question, then, is whether *T cruzi*-induced preferential upregulation of NGF in cultured CFs applies to fibroblasts in their natural myocardium niche.

**Figure 5:**
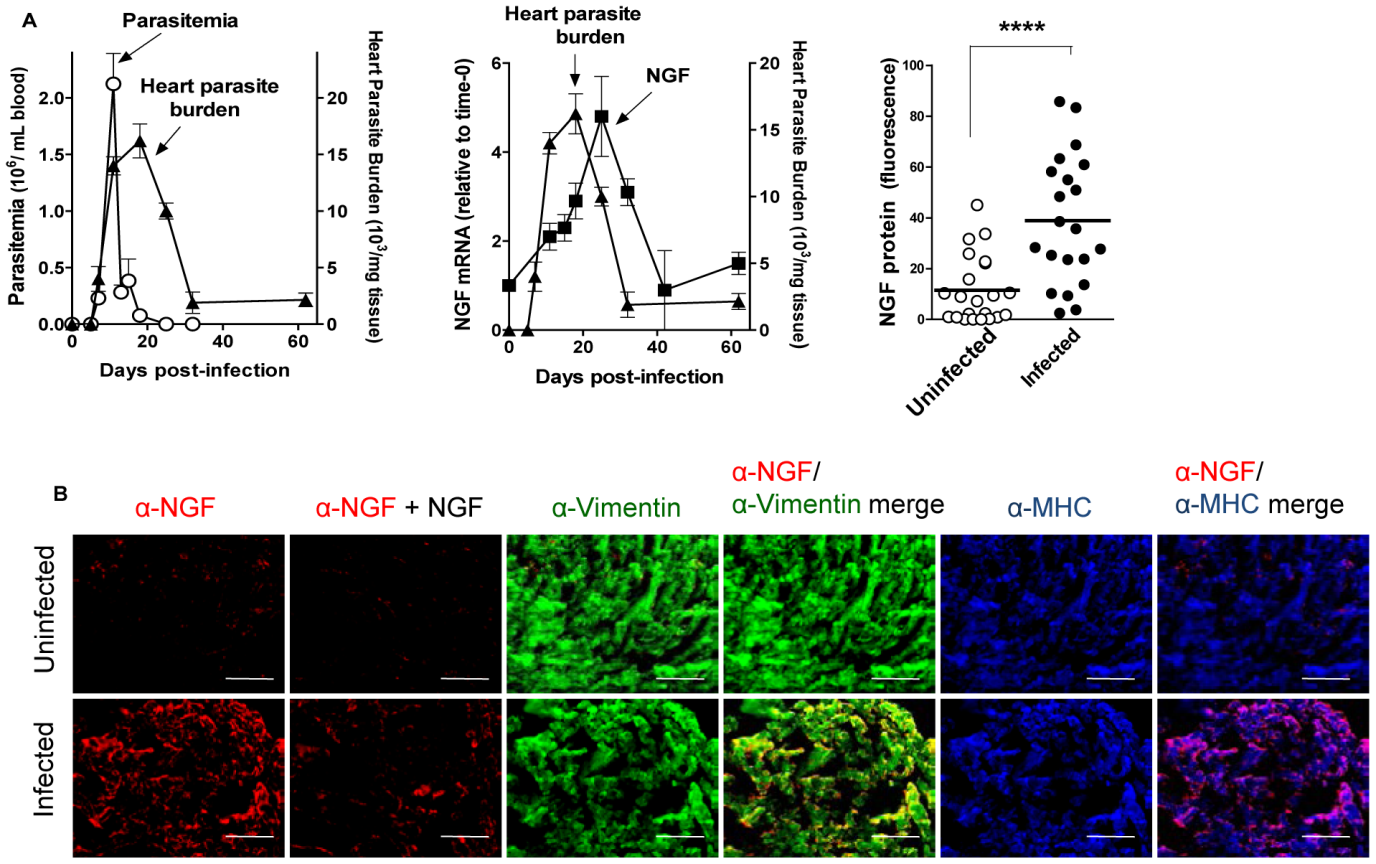
T cruzi infection of the heart upregulate NGF selectively in cardiac fibroblasts. **A**) Upregulation of NGF in the myocardium trails parasitemia and heart parasitism. C57BL/6 mice (2 or 3/point) were infected with T cruzi and their parasitemia, cardiac parasite burden and NGF levels determined at the indicated times post-infection (PI). **Left panel** shows that cardiac parasite burden trails parasitemia. Cardiac parasite burden is a composite of three separate experiments. **Middle panel** shows the kinetics of cardiac NGF transcript in response to T cruzi infection, which trails heart parasitism. **Right panel** displays cardiac NGF protein levels from 20-d PI and vehicle-injected uninfected mice. The results are combined from three independent experiments (n = 22) and show a significant increase in NGF in infected hearts, **** P<0.0001**B**) NGF is preferentially localized in cardiac fibroblasts of T cruzi-infected hearts. The images are representative from the results presented in (**B**), right panel. Note the specificity of NGF visualization, as it is nearly completely blocked by preincubating the NGF antiserum with purified soluble NGF (200 ng/ml) (compare first and second bottom panels, from the left); and the preferential co-localization of NGF on cardiac fibroblasts (yellow color in the α-NGF/α-vimentin merge); scale bars = 100 µm.

We infected C57BL/6 mice with *T cruzi* (Tulahuen strain) and measured the kinetics of parasitemia (by phase-contrast microscopy), heart parasite burden (by qPCR), and cardiac NGF transcript (by qPCR). Parasitemia peaks 11 days PI and heart parasitism 7 days later, a time when parasitemia is no longer detectable (Fig. 5A, left panel). NGF mRNA increases in infected hearts maximally 22–24 d PI, trailing heart parasite burden (Fig. 5A, middle panel), supporting the premise from *in vitro* experiments that NGF upregulation results from *T cruzi* recognition of fibroblast-TrkA. Upregulation of NGF transcript is reflected in NGF protein, which increases in infected hearts as quantified with NIH ImageJ software in tissue sections obtained at peak heart parasitism (20-d PI) and stained by indirect immunofluorescence (Fig. 5A, right panel). Visualization of the fluorescence also reveals a sharp NGF upturn in *T cruzi*-infected hearts (Fig. 4B, leftmost panels). Early studies showed increased NGF in *T cruzi*-infected hearts [43].

To determine whether the observed fluorescence is due to antibody recognition of NGF and not to nonspecific staining, we preincubated the NGF antibody with a low concentration of NGF, which eliminates NGF visualization almost completely (Fig. 5B, second panels from the left), establishing the specificity of NGF detection. Co-staining analysis reveals that NGF merges preponderantly with vimentin (yellow) (Fig. 5B, α-NGF/α-vimentin merge panels), confirming the preferential increase in NGF in the CFs of chagasic hearts.

### The *T cruzi* Trk-ligand PDNF Upregulates NGF Preferentially in Cardiac Fibroblasts *In Vitro* and in the Heart Following Intravenous Administration

Given that *T cruzi* upregulates NGF in cardiac fibroblasts through TrkA, which in turn is recognized by *T cruzi* via PDNF [26], then PDNF must be the *T cruzi* ligand mediating the NGF-stimulatory effect. This prediction was tested in CFs and myocytes in culture and in the myocardium.

PDNF consists of a N-terminal region, containing the catalytic [44] [44] and Trk-binding [26], [27], [28], [31] sites, and a C-terminal long terminal repeat [45], [46], originally identified as shed acute phase antigen (SAPA) [47]. Here we used a short-form of PDNF (sPDNF), devoid of SAPA, recombinantly expressed in bacteria as a soluble protein [31] that retains TrkA/TrkC binding [27], [28].

**Figure 6:**
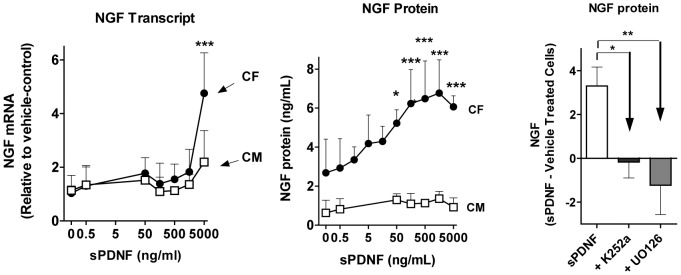
The T cruzi Trk-ligand sPDNF preferentially upregulates NGF in cardiac fibroblasts requiring TrkA signaling. **Left panel**: sPDNF upregulates NGF transcripts in cardiac fibroblasts. Primary cultures of cardiac fibroblasts (CFs) and cardiomyocytes (CMs) were stimulated with the indicated concentrations of recombinant sPDNF for 3 h and their NGF transcript measured by qPCR. **Middle panel**: sPDNF upregulates NGF protein in cardiac fibroblasts. Cultures of CFs and CMs were stimulated with the indicated concentrations of sPDNF for 3 h and their culture overlays assessed for NGF by ELISA. Data are combined from three independent experiments; * P<0.05, *** P<0.001. **Right panel**: An inhibitor of TrkA autophosphorylation and another of TrkA downstream signaling block sPDNF-induced NGF upregulation in cardiac fibroblasts. Primary cardiac fibroblasts (triplicate wells) were pretreated for 30 min with vehicle or with TrkA autophosphorylation inhibitor K252a (500 nmol/L), or with the Erk kinase signaling inhibitor UO126 (10 µmol/L) (+K252a and U0126, respectively) and then with vehicle or sPDNF (500 ng/mL) for 24 h; NGF was measured in culture supernatants by ELISA, results are mean + s.d. and represent the difference between sPDNF and vehicle treated cardiac fibroblasts; * P<0.05, ** P<0.01.

We find that, in fibroblasts, sPDNF stimulates NGF transcript (Fig. 5, left panel) and protein (Fig. 5, middle panel) quickly (3-h post-injection) and dose-dependently. Like *T cruzi*, sPDNF is ineffective in upregulating NGF in primary cardiomyocytes (Fig. 6, left and right panels). Furthermore, and in concert with the use of TrkA by *T cruzi* to enhance NGF production (Fig. 4), the TrkA autophosphorylation inhibitor K252a completely reverses the NGF stimulatory effect of sPDNF, as does the TrkA downstream (Mek1/2 kinases) signaling inhibitor U0126 (Fig. 6, right panel). This indicates that sPDNF requires TrkA signaling to increase NGF production in CFs.

Prior to addressing whether sPDNF augments NGF expression *in vivo*, we obtained preliminary pharmacokinetics parameters by measuring sPDNF decay in the blood and myocardium following intravenous (iv) administration. Tissue sPDNF was quantified by its intrinsic trans-sialidase activity (absent in mammalian tissues) using a ^14^C-based assay [48]. sPDNF has a half-life of ∼15 min in the blood, gains access to the atria and ventricle maximally at least 15 min post-administration, and remains in the heart for >30 min (Fig. S2A). Assuming negligible loss of trans-sialidase activity in tissues after iv injection, the amount of sPDNF uptake by the heart after a single dose is very small, or ∼1/500^th^ the amount of injected sPDNF (Fig. S2B).

**Figure 7:**
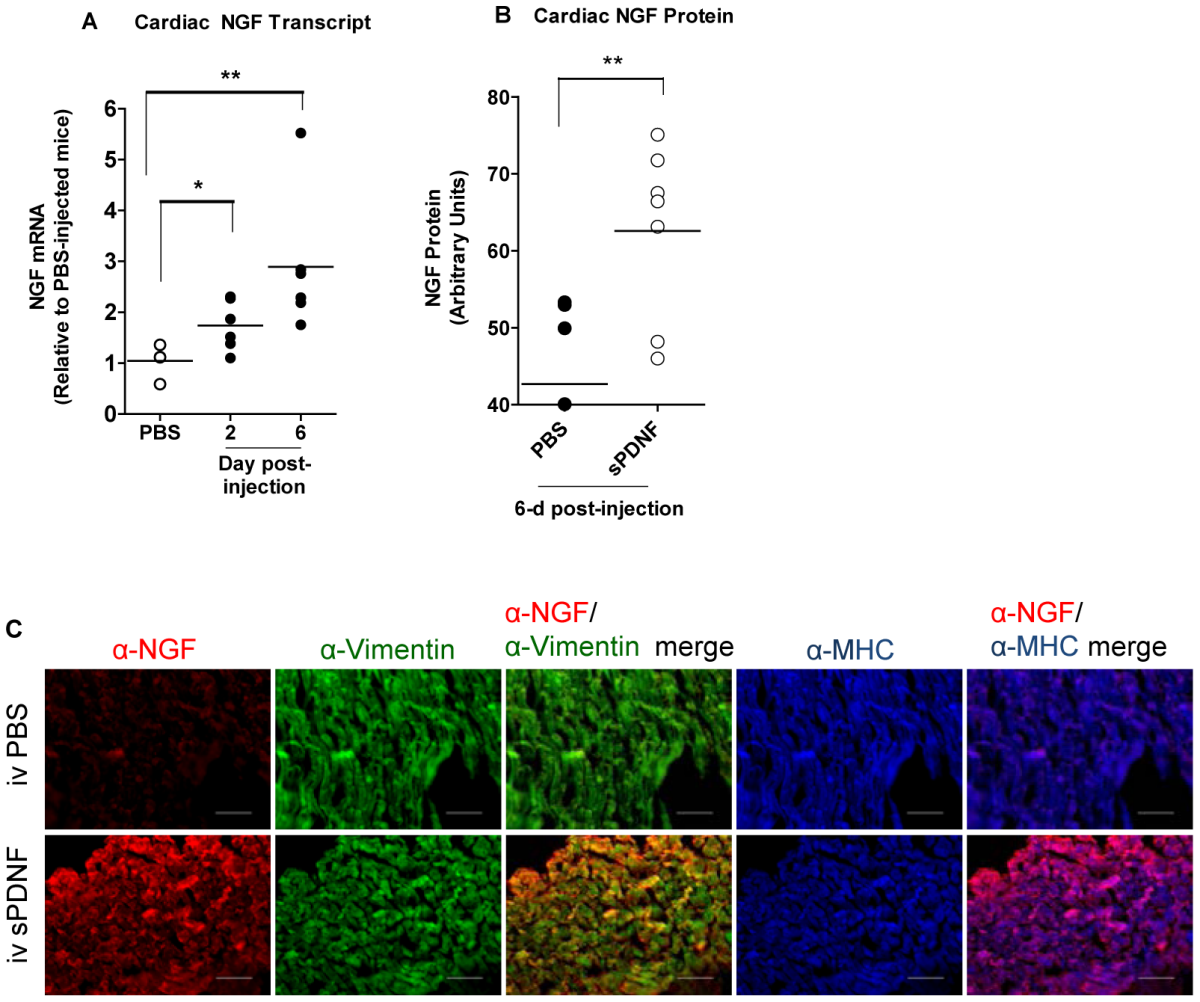
Intravenous administration of PDNF upregulates nerve growth factor in the heart selectively on cardiac fibroblasts. **A**) Intravenous sPDNF upregulates NGF transcript in the heart of C57BL/6 mice. Mice (2–3/point) were injected with vehicle (PBS) or sPDNF intravenously (IV) (25 µg per mouse) daily for 2 or 6 days, sacrificed one day after the last injection, and their cardiac (atria) NGF transcript levels measured in duplicate by qPCR and compared to mice injected with IV PBS for 6 d. Results are representative of three similar experiments; *, P<0.05; ** P<0.01, ns, not statistically significant. **B**) Intravenous sPDNF upregulates NGF protein in the heart of C57BL/6 mice. Mice (2–3/point) were injected with sPDNF intravenously (25 µg per mouse) or vehicle for 6 d, sacrificed one day after the last injection, and their cardiac (atria) NGF protein levels ascertained by immunofluorescence using the NIH ImageJ software, n = 3–7, composite of two distinct experiments. **C**) NGF is preferentially localized in cardiac fibroblasts following intravenous injection of sPDNF. Representative images of heart (atria) tissue sections from the results presented in **B**) above. Note that iv sPDNF robustly increase NGF (leftmost panels) that co-localizes primarily with the cardiac fibroblasts (α-NGF/α-vimentin merge).

Yet, despite low levels in cardiac tissues, a single iv dose (8.3 mg/Kg) of sPDNF ups cardiac NGF mRNA as early as 3-h post-injection (Fig. S3). Daily iv injections of a lower sPDNF dose (1.4 mg/Kg) for 2 or 6 days also increases cardiac NGF transcript (Fig. 7A) and protein (Fig. 7B). As in chagasic hearts, NGF increases selectively in CFs following iv sPDNF, as indicated by the merge of NGF, stained red, with vimentin, stained green, giving rise to yellow-merged color (Fig. 7C, α-NGF/α-vimentin merge panels). Fluorescently labeled NGF does not appear to merge with the cardiomyocyte marker MHC (Fig. 7, α-NGF/α-MHC merge panels). To confirm immunofluorescence results, we measured NGF protein by ELISA in heart extracts following i.v. sPDNF and found increased levels as early as two hours post-injection (Fig. S4). These findings indicate that *T cruzi* upregulates NGF *in vitro* and *in vivo* through PDNF.

### Conditioned Media Obtained from *T cruzi*-Infected or sPDNF-Stimulated Cardiac Fibroblasts Confer Cardiomyocyte Protection against Oxidative Stress

We used conditioned medium (CoM) and un-conditioned medium (Un-CoM), obtained from primary cultures of infected with *T cruzi* (24 h) or not, respectively, to determine if the *T cruzi-*CF interaction triggers cardiomyocyte survival via paracrine signaling. CoM and Un-CoM were centrifuged (2, 000×g, 10 min) and filtered through 0.22 µ pores prior to use. Then, we assessed the protective effect of CoM in cardiomyocytes exposed to cardiotoxic H_2_O_2_ using the propidium iodide and vital stain Hoechst 33342 procedure.

**Figure 8:**
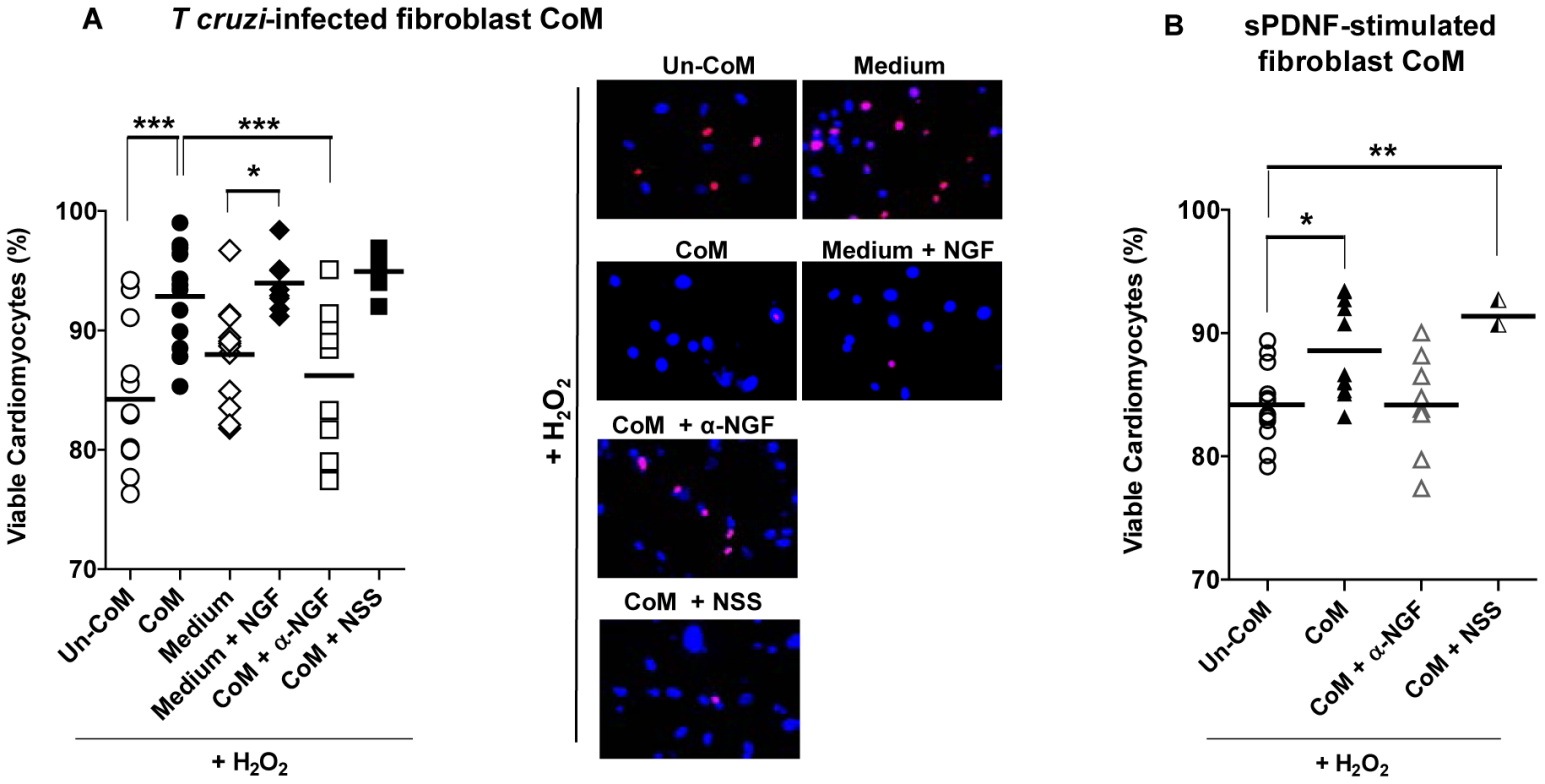
Conditioned media from T cruzi-infected or sPDNF-stimulated cardiac fibroblasts protect cardiomyocytes against oxidative stress. **A**) Conditioned media produced by T cruzi infection of cardiac fibroblasts confer protection of cardiomyocytes against H_2_O_2_-induced death in an NGF-dependent manner. Primary cultures of cardiomyocytes were exposed to 150 µM H_2_O_2_ for 4 h following preincubation with unconditioned medium from control fibroblast cultures (Un-CoM) or conditioned medium obtained by infecting cardiac fibroblasts with T cruzi (2×10^5^/mL, 24 h) (CoM). Cardiomyocytes were also placed in medium unexposed to cells, with or without 50 ng/mL NGF. Lastly, CoM was mixed with an α-NGF-blocking sheep antiserum (α-NGF) or normal sheep serum (NSS). Panel on the right shows representative staining from each condition. Data are combined from three independent experiments. **B**) Conditioned media produced by the specific stimulation of cardiac fibroblast by sPDNF confer protection of cardiomyocytes against H_2_O_2_-induced death in an NGF-dependent manner. Cardiomyocytes were exposed to 150 µM H_2_O_2_ for 4 h following preincubation with unconditioned medium (Un-CoM) or conditioned media obtained by stimulating cardiac fibroblasts with sPDNF (50 ng/ml, 3 h) (CoM). Cardiomyocytes were also exposed in parallel to CoM preincubated with an α-NGF-blocking sheep antiserum (α-NGF) or normal sheep serum (NSS). Data are combined from three independent experiments; * p<0.05, **p<0.01 ***p<0.001.

Compared to un-CoM and medium, CoM significantly (P<0.001) reverses the death-causing effect of H_2_O_2_ (Fig. 8A, scatter plot). Exogenous NGF (50 ng/ml) also protects cardiomyocytes against H_2_O_2_ in this assay (Fig. 8A; compare medium with medium + NGF). Furthermore, a neutralizing NGF serum (α-NGF) significantly (P<0.001) inhibits the protective action of CoM under conditions control normal serum (NSS) does not (Fig. 8A, scatter plot). This indicates that NGF is at least one of the cardioprotective factors that *T cruzi* induces cardiac fibroblasts to secrete into the extracellular milieu (i.e., CoM). The panel on the right of Fig. 8A displays representative staining of each of these conditions.

Fitting perfectly well with the concept that *T cruzi,* via PDNF, augments NGF secretion in CFs, CoM produced by stimulating CFs at low sPDNF concentration and for a short time (50 ng/ml, 3 h) significantly protects cardiomyocytes against H_2_O_2_-induced cell death, a protection abrogated by a neutralizing NGF antiserum (Fig. 8B).

## Discussion

Our results demonstrate, for the first time, that primary neonatal CFs express transcripts of NGF/TrkA and NT-3/TrkC pairs, and of BDNF, a pattern similar to that of primary cardiomyocytes except for the very low/undetectable expression of NT-3 in the myocytes (Figs. 1A and B). NGF had the highest expression of the three in both adult and neonatal cardiac cells, whereas BDNF expression declined and NT-3 expression increased from neonatal to adult cells.

NGF is the most studied NT in cardiac injury. Earlier findings showed that NGF, which signals via TrkA but not TrkB and TrkC [2], triggers prosurvival activity in primary cardiomyocytes through an autocrine mechanism [10]. This activity may be beneficial *in vivo*, as NGF gene therapy is cardioprotective in models of myocardial infarction [12] and diabetes [13]. However, those *in vivo* studies were not designed not tell whether myocardial NGF secreted under physiological conditions and/or in response to stressors arise from cardiomycytes. Furthermore, although it has been established that cardiac injury, such as in hypoxemia/reperfusion or myocardial infarction, sharply augments cardiac NGF [7], [12], [13], nothing is known of the ligands and cell-surface recepor(s) that trigger NGF upregulation, except for endothelin-1, which regulates NGF expression in cardiomycytes [8]. However, it remains unknown whether endothelin-1 alters cardiac NGF expression in fibroblasts, which express endothelin-1 receptors [49]. Identification of the molecules that control NGF upregulation is clearly important to better understand cardiac function, response to injury, and design therapeutics to boost myocardial NGF, including by systemic administration.

*T cruzi* invades the heart where it causes acute myocarditis that lasts a few months, followed by a chronic debilitating cardiomyopathy in ∼30% infected individuals many years after the infection [24], [25]. Therefore, the possibility exists that *T cruzi*/host interaction triggers reactions that reduce or prevent damage of infected tissues. This idea is backed up by our findings presented here, which demonstrate that *T cruzi* triggers an exuberant production of cardioprotective NGF (more than 80-fold increase) in cardiac cells growing in culture, predominantly in fibroblasts (Fig. 3). This NGF boost response discriminates between *T cruzi* recognition of TrkA and TrkC, as it requires the former but not the latter (Fig. 4), an *in vitro* response analogous to that seen in chagasic hearts (Fig. 5). Reflecting the *T cruzi* recognition of Trks via its PDNF, bacterially expressed PDNF (sPDNF) reproduces the effect of *T cruzi* infection, for it selectively boosts NGF in fibroblasts outside (Fig. 6) and inside their natural myocardial niche (Fig. 7).

Given the prosurvival action of NGF on cardiomyocytes and the vigorous upregulation of bioactive NGF selectively in fibroblasts in response to *T cruzi* infection or sPDNF stimulation, it is reasonable to hypothesize that *T cruzi*-cardiac fibroblast interaction protects cardiomyocytes via a NGF-dependent paracrine mechanism. This prediction proved to be correct as medium conditioned by *T cruzi* infection or sPDNF stimulation of fibroblasts prevented cardiomyocyte death resulting from oxidative stress (hydrogen peroxide) (Fig. 8). It has long been known that oxidative stress kills cardiomyocytes [50], including in chagasic cardiomyopathy development [51].

Furthermore, NGF released following *T cruzi*-fibroblast interaction may also protect cardiomyocytes against deleterious effects additional to the oxidative stress shown here, as judged by the findings of others demonstrating NGF protection of cardiomyocytes subjected to growth factor deprivation, hypoxia/reoxygenation, and angiotensin II stimulation [12]. We therefore hypothesize that enhanced NGF secretion following *T cruzi-*PDNF recognition of fibroblast-TrkA is a mechanism responsible for long term symtomless and relatively pathology-free *T cruzi* homing in the heart.

It will be of importance to know the biological outcome of *T cruzi* binding to TrkA or TrkC on cardiomyocytes, and TrkC on fibroblasts. It will also be important to know whether endogenous TrkA-binding molecules such as NGF reproduce the *T cruzi* action in fibroblasts.

Finally, as PDNF binding to TrkA on fibroblasts releases NGF (Fig. 6) which, in turn, protects cardiomyocytes against oxidative stress (Fig. 8B) and perhaps other insults [10], and, as intravenous administration of sPDNF boosts cardiac NGF (Fig. 7), our results offer a possible novel translational medicine opportunity for cardiomyopathies where NGF gene therapy has proven to be valuable in myocardial infarction [12] and diabetes [52]. PDNF administration might also be useful in preventing or slowing down cardiomyocyte degeneration. PDNF might be a better therapeutic than NGF because it 1) activates both prosurvival TrkA and TrkC while NGF activates only TrkA; 2) has a unique dual prosurvival activity by directly activating TrkA and TrkC outside cells and, in the intracellular milieu, pro-survival Akt kinase [33]; 3) creates a positive TrkA signaling loop by robustly upregulating NGF; 4) has a relatively long half-life (detectable in the heart for >30 min after iv injection, see Fig. S2).

## Supporting Information

Figure S1 **sPDNF pharmacokinetics in the heart following intravenous administration.** **A**) Dissociation curves of qPCR reactions using SYBR green detector. Two samples each of adult cardiac fibroblast and cardiomyocyte cDNA were probed for the indicated genes and dissociation curves are displayed. **B**) qPCR products for HPRT, Trk receptors, and NTs were run on an ethidium-bromide stained 1.5% agarose gel to demonstrate homogeneity of reactions. Bottom two bands of ladder are 100 and 200 base pairs (bp) for comparison and expected product sizes for each amplicon are listed below gel.
(TIF)

Figure S2 **sPDNF pharmacokinetics in the heart following intravenous administration.** sPDNF was injected at 1.4 mg/kg (or 25 µg per mouse) into female C57BL/6 mice (5/group), and the mice sacrificed at 15, 30 and 120 min post-injection, perfused with PBS (5 ml), and sPDNF assessed in homogenized atria and ventricles by measuring *trans*-sialidase activity of the cardiac tissues by a C^14^-based assay. **A**) Decay of concentration of sPDNF in tissues as ng/mg tissues; **B**) decay of sPDNF in tissues as % injected sPDNF.
(TIF)

Figure S3 **Intravenous administration of sPDNF increases NGF protein by ELISA.** Tissue extracts from pharmacokinetics experiment (Fig. S2) were tested for NGF concentration by ELISA. Two technical replicates each on duplicate points are graphed as mean + s.d., * P<0.05.
(TIF)

Figure S4 **Intravenous administration of sPDNF ups NGF mRNA as early as three hours post-injection.** C57BL/6 mice (two per point) were injected with 150 µg sPDNF or PBS vehicle medium into their tail veins. After 3, 6, 9, or 12 hours, mice were sacrificed via CO_2_ asphyxiation, and their cardiac NGF transcript quantified by qPCR. Fold expression was calculated using the 2^−ddCt^ method using HPRT as the internal control and PBS-injected mice as the negative control.
(TIF)

Table S1 **Antibodies and concentrations.** Antibody sources and concentrations used in immunofluorescence assays.
(TIF)

Table S2 **qPCR Primers.** Sequences of primers used for qPCR reactions.
(TIF)
