# Supplementary figures and images for: Trypanosoma cruzi Coaxes Cardiac Fibroblasts into Preventing Cardiomyocyte Death by Activating Nerve Growth Factor Receptor TrkA
Source: PLoS One. 2013 Feb 21;8(2):e57450. doi: 10.1371/journal.pone.0057450 (PMC3578799; doi:10.1371/journal.pone.0057450)

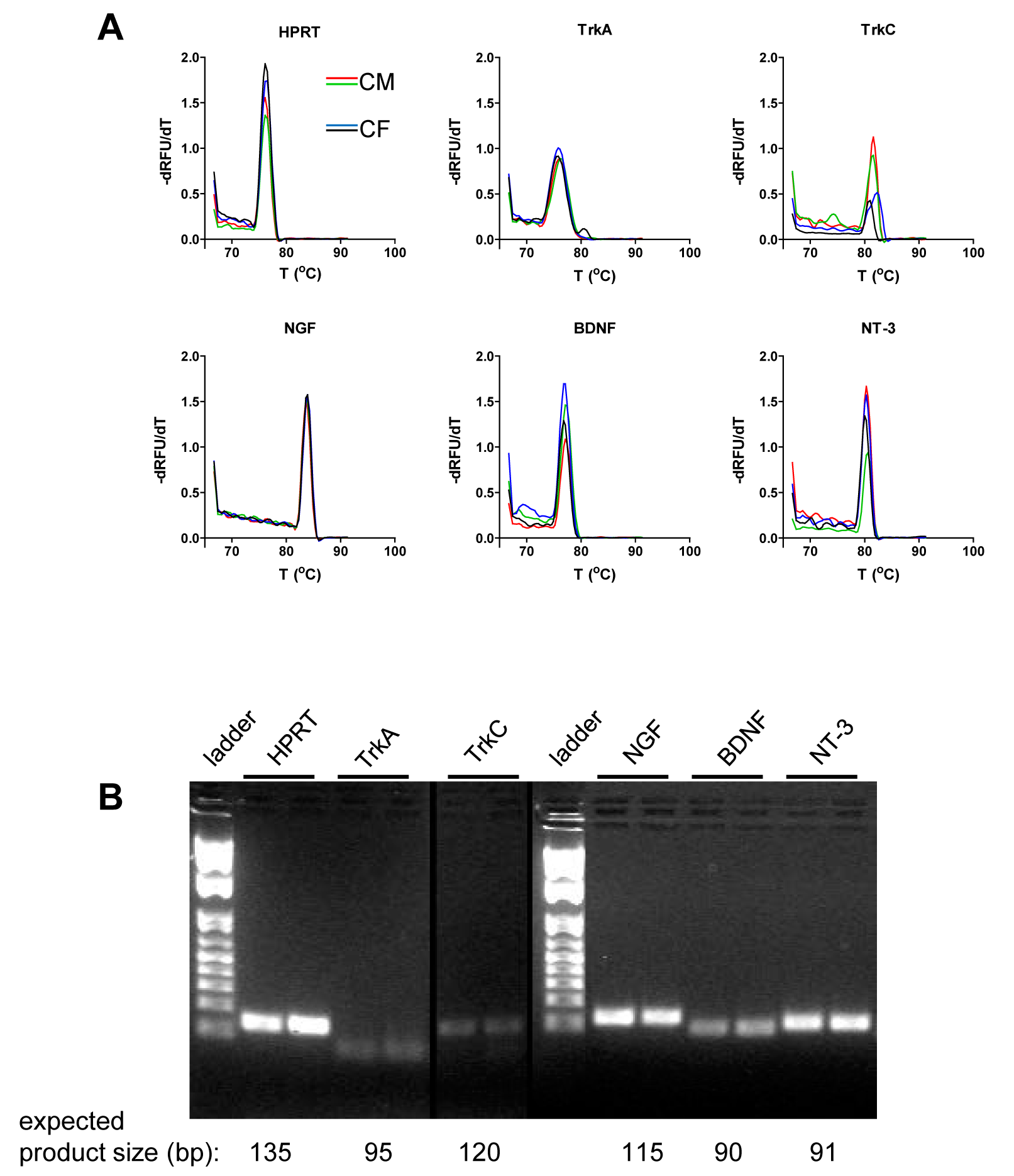

Supplement: Figure S1 — sPDNF pharmacokinetics in the heart following intravenous administration. A) Dissociation curves of qPCR reactions using SYBR green detector. Two samples each of adult cardiac fibroblast and cardiomyocyte cDNA were probed for the indicated genes and dissociation curves are displayed. B) qPCR products for HPRT, Trk receptors, and NTs were run on an ethidium-bromide stained 1.5% agarose gel to demonstrate homogeneity of reactions. Bottom two bands of ladder are 100 and 200 base pairs (bp) for comparison and expected product sizes for each amplicon are listed below gel. (TIF) [file pone.0057450.s001.tif]

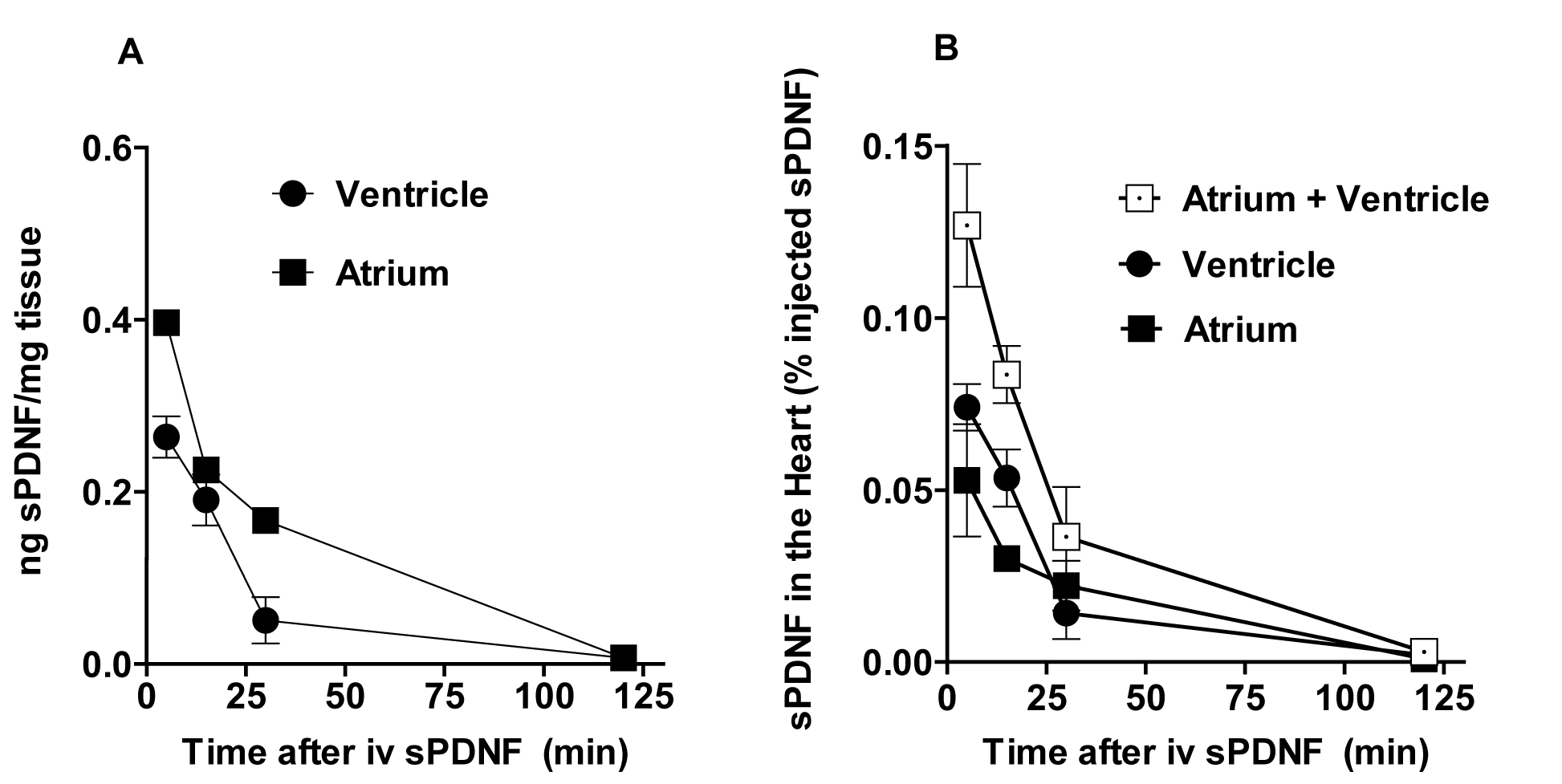

Supplement: Figure S2 — sPDNF pharmacokinetics in the heart following intravenous administration. sPDNF was injected at 1.4 mg/kg (or 25 µg per mouse) into female C57BL/6 mice (5/group), and the mice sacrificed at 15, 30 and 120 min post-injection, perfused with PBS (5 ml), and sPDNF assessed in homogenized atria and ventricles by measuring trans-sialidase activity of the cardiac tissues by a C14-based assay. A) Decay of concentration of sPDNF in tissues as ng/mg tissues; B) decay of sPDNF in tissues as % injected sPDNF. (TIF) [file pone.0057450.s002.tif]

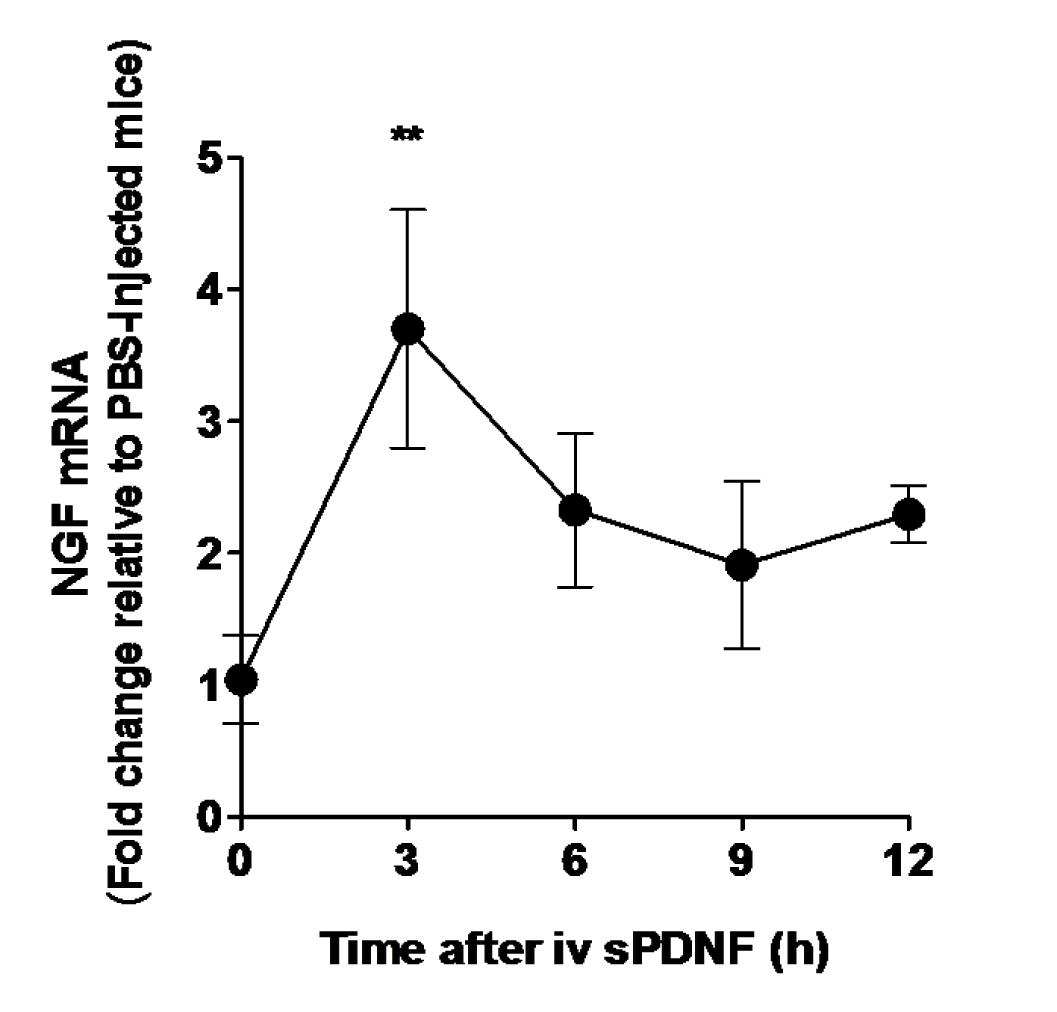

Supplement: Figure S3 — Intravenous administration of sPDNF increases NGF protein by ELISA. Tissue extracts from pharmacokinetics experiment (Fig. S2) were tested for NGF concentration by ELISA. Two technical replicates each on duplicate points are graphed as mean + s.d., * P<0.05. (TIF) [file pone.0057450.s003.tif]

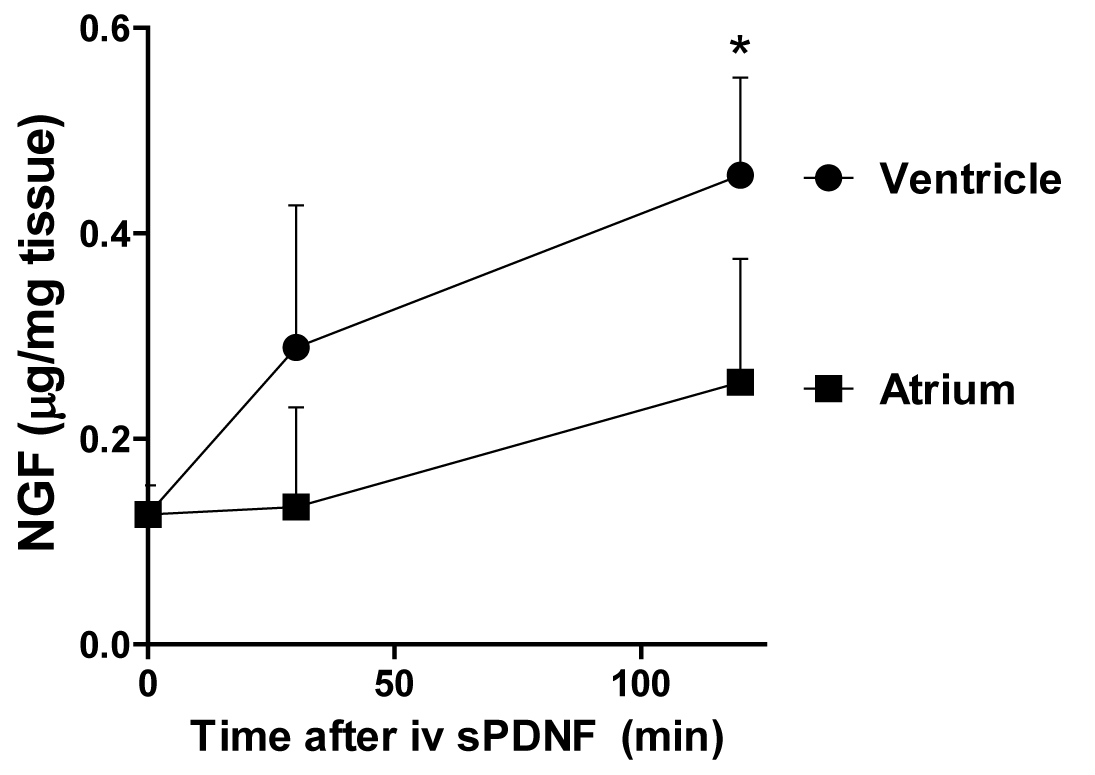

Supplement: Figure S4 — Intravenous administration of sPDNF ups NGF mRNA as early as three hours post-injection. C57BL/6 mice (two per point) were injected with 150 µg sPDNF or PBS vehicle medium into their tail veins. After 3, 6, 9, or 12 hours, mice were sacrificed via CO2 asphyxiation, and their cardiac NGF transcript quantified by qPCR. Fold expression was calculated using the 2−ddCt method using HPRT as the internal control and PBS-injected mice as the negative control. (TIF) [file pone.0057450.s004.tif]

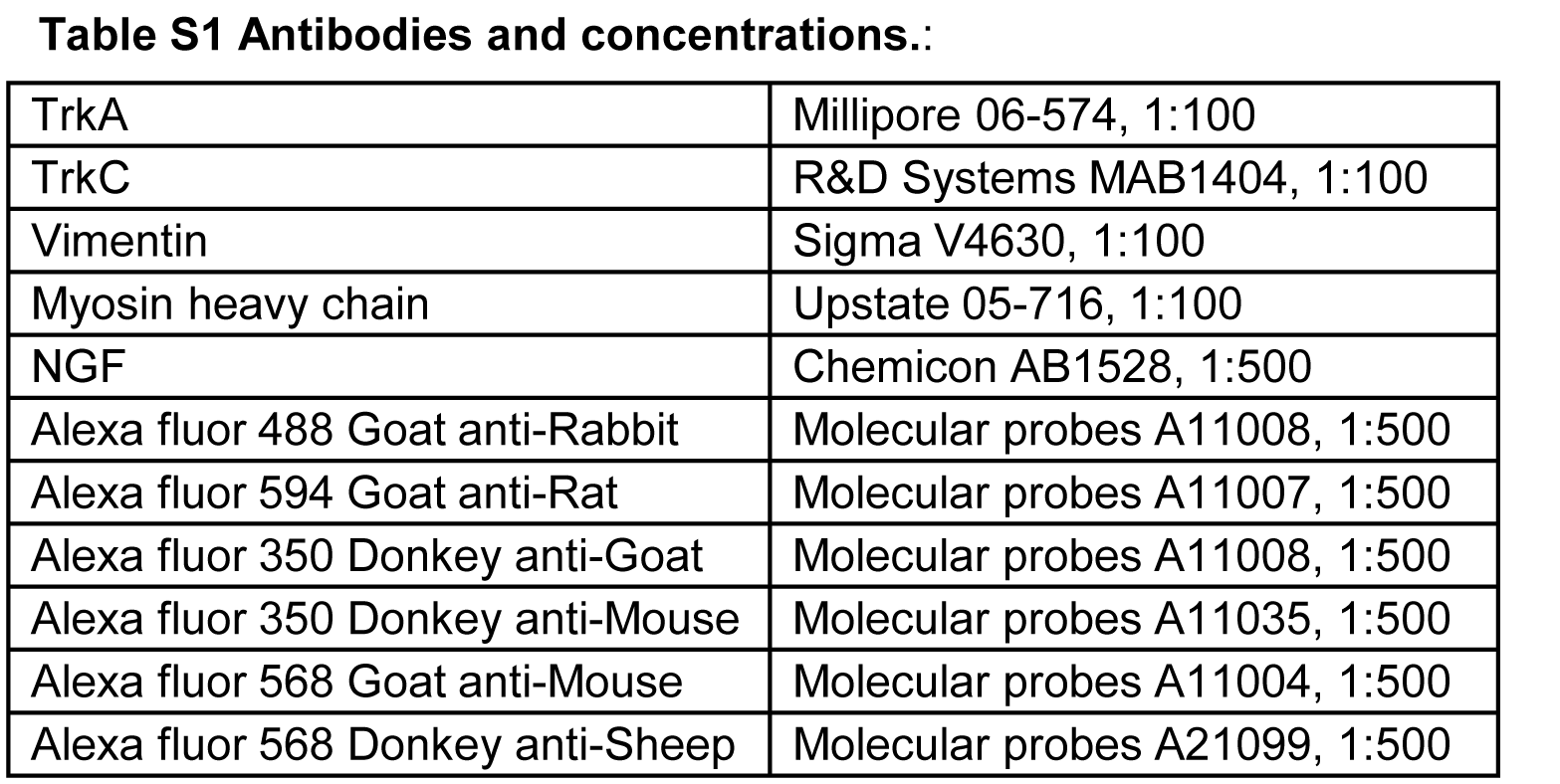

Supplement: Table S1 — Antibodies and concentrations. Antibody sources and concentrations used in immunofluorescence assays. (TIF) [file pone.0057450.s005.tif]

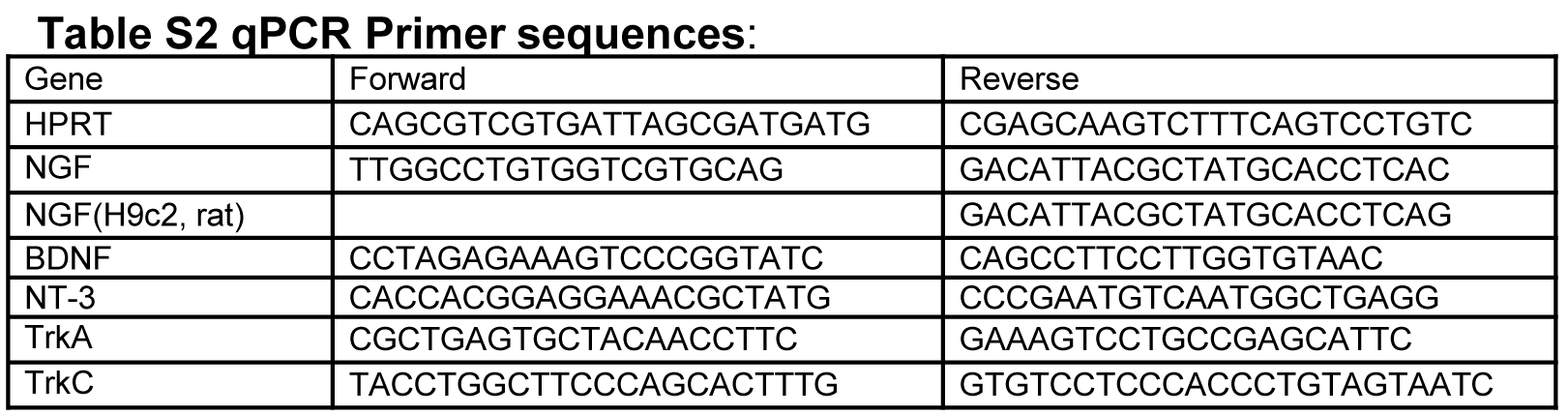

Supplement: Table S2 — qPCR Primers. Sequences of primers used for qPCR reactions. (TIF) [file pone.0057450.s006.tif]
